# DNA Methylation Modifications Associated with Chronic Fatigue Syndrome

**DOI:** 10.1371/journal.pone.0104757

**Published:** 2014-08-11

**Authors:** Wilfred C. de Vega, Suzanne D. Vernon, Patrick O. McGowan

**Affiliations:** 1 Centre for Environmental Epigenetics and Development, University of Toronto, Scarborough, ON, Canada; 2 Department of Biological Sciences, University of Toronto, Scarborough, ON, Canada; 3 Department of Cell and Systems Biology, University of Toronto, Toronto, ON, Canada; 4 CFIDS Association of America, Charlotte, North Carolina, United States of America; Bellvitge Biomedical Research Institute (IDIBELL), Spain

## Abstract

Chronic Fatigue Syndrome (CFS), also known as myalgic encephalomyelitis, is a complex multifactorial disease that is characterized by the persistent presence of fatigue and other particular symptoms for a minimum of 6 months. Symptoms fail to dissipate after sufficient rest and have major effects on the daily functioning of CFS sufferers. CFS is a multi-system disease with a heterogeneous patient population showing a wide variety of functional disabilities and its biological basis remains poorly understood. Stable alterations in gene function in the immune system have been reported in several studies of CFS. Epigenetic modifications have been implicated in long-term effects on gene function, however, to our knowledge, genome-wide epigenetic modifications associated with CFS have not been explored. We examined the DNA methylome in peripheral blood mononuclear cells isolated from CFS patients and healthy controls using the Illumina HumanMethylation450 BeadChip array, controlling for invariant probes and probes overlapping polymorphic sequences. Gene ontology (GO) and network analysis of differentially methylated genes was performed to determine potential biological pathways showing changes in DNA methylation in CFS. We found an increased abundance of differentially methylated genes related to the immune response, cellular metabolism, and kinase activity. Genes associated with immune cell regulation, the largest coordinated enrichment of differentially methylated pathways, showed hypomethylation within promoters and other gene regulatory elements in CFS. These data are consistent with evidence of multisystem dysregulation in CFS and implicate the involvement of DNA modifications in CFS pathology.

## Introduction

Chronic Fatigue Syndrome (CFS), also known as myalgic encephalomyelitis, is a complex multifactorial disease that is characterized by an unexplained fatigue lasting for a minimum of 6 months as well as the presence of at least 4 of the following symptoms: muscle or joint pain, lack of refreshing sleep, headache, sore throat, post-exertional malaise, tender cervical and axillary lymph nodes, and impaired memory and concentration [1]. The symptoms fail to dissipate after sufficient rest and have a clear effect on daily functioning. CFS has an estimated economic impact of $9.1 billion USD in lost productivity in the United States [2]. The biological basis of CFS remains poorly understood. Substantial heterogeneity in symptoms exists among patient populations diagnosed with CFS, suggesting that CFS dysfunctions may involve multiple systems, including neuroendocrine, autonomic, metabolic and neurobiological [3]–[5]. However, symptoms linked to immune dysregulation and abnormalities in immune system function are a consistent feature of CFS [6].

Studies examining gene regulation using whole blood and peripheral blood mononuclear cells (PBMCs), composed primarily of lymphocytes and monocytes, point towards abnormalities in lymphocyte function in CFS. CFS sufferers exhibit disrupted homeostasis between the Th1- (cell-mediated) and Th2- (humoral) immune response, where CFS is associated with a predominantly Th2-mediated immune response [7], [8]. This shift towards Th2-responses is accompanied by reported increases in anti-inflammatory cytokines in CFS [7], [9], [10]. However, cytokine profile changes in CFS remain unclear, as other microarray and cytokine profiling studies have found evidence of increased pro-inflammatory cytokine expression in CFS [11], [12]. It has been reported that natural killer cells show impaired function in CFS [8], [13]–[15]. A difference in CD8+ T cell activation is also a prevalent finding among studies [11], [12], [15]–[17]. Thus, it remains unclear what immune cell type is most relevant in CFS pathology, and discrepancies in immunological results could be explained by study parameters such as methodological differences, as well as heterogeneity in clinical characteristics linked to CFS [12], [13], [15]–[19].

An accumulating number of studies have examined epigenetic modifications associated with immune responses in the context of disease [20], [21]. Epigenetic modifications such as DNA methylation, which mainly occurs on the cytosines of CpG dinucleotide sites (CpG) across the genome, may regulate gene expression without a change in the underlying gene sequence and arise through genetic, stochastic, and environmental factors [22]. To our knowledge, epigenomic changes associated with CFS have not been explored.

For this exploratory study, we selected 12 female CFS patients and 12 healthy control females from a total of 231 patients recruited from 4 clinical centers in order to match CFS patients and control subjects for age and body mass index (BMI), excluding obese subjects and subjects with a history of exposure to immunomodulatory medications, as these conditions may alter epigenetic and immune profiles [23]–[25]. Methylomes in PBMCs were examined using the Illumina HumanMethylation450 BeadChip (450 K) array, which offers coverage of more than 480,000 CpG sites and 98.9% of RefSeq genes in the human genome [26]. We performed gene ontology (GO) and gene network analysis on differentially methylated genes in order to determine biological pathways associated with methylation changes in CFS.

## Materials and Methods

### Ethics statement

This study adhered to the human experimentation guidelines as outlined by the Helsinki Declaration of 1975. The collection and analysis of clinical information and biological samples by the SolveCFS BioBank was ethically approved by the University of Toronto (IRB #27391) and the Genetic Alliance ethics review board (IRB # IORG0003358), which approved all procedures for obtaining written informed consent from all subjects to participate in this study. Consent forms were signed in duplicate, with one copy provided to subjects and one copy securely stored at the SolveCFS Biobank.

### Subjects and selection criteria

Volunteers diagnosed with CFS and healthy controls were recruited by the SolveCFS BioBank. Comprehensive medical histories of the volunteers were recorded, including demographic data, age of CFS onset (if diagnosed with CFS) and medication use. The RAND-36, a validated 36 item self-report inventory, was used to assess health-related quality of life [27]. CFS diagnosis was based on the Fukuda and the Canadian criteria [1], [28]. Volunteers with AIDS, HIV, and/or Hepatitis C were initially screened out. We recruited 231 volunteers from 4 clinical centers. For this exploratory study, we selected white female subjects, 52 years of age or younger, non-obese (BMI<30) and with no previous consumption of immunomodulatory medications and medications with known effects on epigenetic mechanisms (Table S1), to exclude factors associated with epigenetic differences and altered immune profiles [23]–[25]. All CFS subjects had an infectious phenotype where subjects reported the onset of flu-like illness prior to CFS diagnosis. After applying these exclusion criteria, 12 CFS patients and 12 healthy controls matched for age (within the range of 23–52 years old) and BMI (within the range of 18.6–29.8) were available for DNA methylation analysis.

### Blood collection and PBMC isolation

Whole blood in heparanized tubes collected at 4 clinical sites was sent to the Rutgers University Cell and DNA Repository at ambient temperature via overnight shipping. Plasma was collected after centrifugation and the remaining blood was diluted with DPBS. Processing of samples followed guidelines approved by Rutgers University (Newark, NJ). Briefly, PBMCs were isolated using Ficoll gradient centrifugation and resuspended in 1X DPBS +1% fetal bovine serum. A sample was taken for automatic cell counting using a ViCell XR Viability Analyzer. Dry pellets of 10×10^6^ cells were stored at –80**°**C after centrifugation.

### Genomic DNA extraction and preparation

For each subject, genomic DNA was extracted from approximately 2.50×10^6^ cells using the Omega E.Z.N.A. Tissue DNA Kit (Omega Bio-Tek, cat. no. D3396) according to the manufacturer’s instructions. Genomic DNA was eluted in Tris-EDTA buffer (10 mM Tris-CL, pH 8.5, 1 mM EDTA) and a NanoDrop 2000c spectrophotometer (Thermo Scientific, Waltham, MA, USA) was used to check the quantity and quality of the DNA. All samples were prepared to a minimum final concentration of 100 ng/µl, A_260_/A_280_ = 1.8–2.0, and A_260_/A_230_>2.0. When required, DNA cleanup was performed using the MinElute Reaction Cleanup Kit (Qiagen Canada, cat. no. 28204) according to the manufacturer’s instructions.

### Epigenomic microarray data collection

Approximately 1.5 µg of DNA was sent to Genome Québec (Montréal, QC, Canada) on dry ice. Samples were processed according to Genome Québec and Illumina protocols for the Infinium HumanMethylation450 BeadChip (450 K) array. This microarray offers coverage of more than 480,000 CpG methylation sites including promoters, untranslated regions (UTRs), first exons, gene bodies, and CpG islands. Annotations in relation to genic regions and CpG islands were based on annotation available from the UCSC Genome Browser (http://genome.ucsc.edu/), where CpG island Shores were defined as the 2 kb regions immediately upstream (North) or downstream (South) of a CpG island, and CpG island Shelves were defined as the 2 kb regions immediately upstream or downstream of a CpG island shore [26]. The raw data have been deposited in Gene Expression Omnibus (GEO) at NCBI (www.ncbi.nlm.nih.gov/geo/) under the accession number GSE59489.

### Microarray validation

Three sites that were identified as differentially methylated between CFS patients and healthy controls were selected for pyrosequencing based on validated primer availability from EpigenDX (Hopkinton, MA, USA). Extracted DNA was sodium bisulfite converted using the EZ DNA Methylation Kit according to the manufacturer’s protocol (Zymo Research, cat. no. D5001). EpigenDX performed pyrosequencing with the following primer sets: HIPK3 (ADS1419-FS2), LCN2 (ADS1074-FS2), LY86 (ADS2397-FS).

### Gene Ontology (GO) and network analysis

An analysis examining all GO terms was performed with DAVID (http://david.abcc.ncifcrf.gov/) using differentially methylated probes as the gene list and the 450 K Illumina microarray as the background. This analysis examines the statistical association between genes in the gene list provided and GO terms and determines the enrichment of GO terms relative to the background gene set, as performed in several previous epigenomic studies using the Illumina methylation microarrays [29]–[34]. Only GO terms were considered when generating DAVID results, as GO terms are consistently updated and allow for comparison across GO analysis platforms. The DAVID results of the differentially methylated gene list were visualized in Enrichment Map to generate a network map of GO terms. Details of how Enrichment Map generates network maps from GO results are described in Merico et al. [43]. Briefly, GO terms are represented as nodes, where node size is related to the number of genes within each GO term, and edges between nodes represent common genes, where edge thickness is proportional to the number of common genes. Overlap between GO terms is calculated by the overlap coefficient, which best handles hierarchically-organized gene set collections [43]. Enrichment Map aims to reduce the redundancy found within typical GO results by grouping similar GO terms together. Clusters that form within the network aid in the delineation of themes associated with the GO results, facilitating biological interpretation of the significant GO terms associated with the differentially methylated gene list. Clusters for the DAVID results were arranged using the yFiles Organic algorithm and named using the WordCloud plugin, which generates a word tag cloud from a user-defined node selection in order to textually summarize biological themes associated with groups of GO terms (http://baderlab.org/Software/WordCloudPlugin). GO analysis was also performed with the GeneMANIA plugin in Cytoscape 2.8.2 (http://www.genemania.org/plugin/) using the 2012-08-12 human genome GeneMANIA build as the background. An application with similar functionality to WordCloud was not available for the GeneMANIA results.

Genes from all GO clusters and the immune cell regulation cluster with differentially methylated CpG sites were extracted for further analysis. Differentially methylated CpG sites from each of these genes were examined according to methylation status (hyper- and hypomethylated based on positive and negative beta-difference values respectively), genic location, location relative to a CpG island, and according to the 3 broad functional categories: gene promoters within 1500 bp and 200 bp of the transcription start site (TSS: TSS1500, TSS200), regulatory elements (regulatory: TSS1500, TSS200, 5′ UTR, 3′ UTR), and within the coding regions of genes (gene body).

### Statistical Analyses

DNA methylation analysis was performed using R software with the Illumina Methylation Analyzer (IMA) package [35]. Methylation values for each of the probes on the 450 K microarray were produced as beta-values, calculated as the ratio of methylated probe intensity over total intensity (methylated and unmethylated) for each probe, which range from 0 to 1 and roughly corresponds to the methylation percentage of the probe [26]. The data was quantile normalized and peak-corrected [36], [37], and low quality probes (detection p-value≥0.01), 92,667 probes overlapping SNPs either at or within 10 bp of the targeted CpG site, and invariable probes with a mean beta-value ≥0.95 or ≤0.05 across case and control samples were removed from analysis [38], [39]. Sites were considered to be differentially methylated if they met the following selection criteria: the absolute beta-value difference between the mean beta-values of cases and controls was greater than 0.20, p≤0.05 according to the Wilcoxon rank-sum test, and FDR≤0.05 using the Benjamini-Hochberg procedure [40], following methods used in several previous epigenomic studies [29], [30], [33], [41], [42]. Lists of differentially methylated probes, regions, and their annotations were generated using sitetest, outputDMfunc, regionswrapper, and annotfunc IMA functions.

IBM SPSS software (Version 22) was used to perform statistical analysis on average age and BMI (Student’s t-test), RAND-36 scores (Student’s t-test), and average methylation according to pyrosequencing (Wilcoxon rank-sum test) using Bonferroni correction for multiple comparisons where applicable. GO results from both DAVID and GeneMANIA were considered to be significant if they survived an FDR≤0.10 cutoff. DAVID GO results were clustered using default settings of the Enrichment Map plugin [43] in Cytoscape 2.8.2 which are: p-value cutoff = 0.005, FDR q-value cutoff = 0.1, overlap coefficient cutoff = 0.5, and combined constant = 0.5 to generate a network map. Differences between cluster groups in the relative proportions of hyper- and hypo-methylated CpG sites within each region (TSS, regulatory, gene body) were examined with Pearson’s chi-squared tests, using Bonferroni correction for multiple comparisons.

## Results

### Subject demographics, characteristics, and RAND-36 scores

Table 1 shows the demographics, diagnostic history, and RAND-36 scores of the subjects that met criteria for this study (see Methods). The subject groups did not significantly differ in age and BMI. Several aspects of physical and social functioning were impacted in CFS patients and, as assessed using the RAND-36, were significantly different from matched healthy controls (all p’s<0.05, Student’s t-tests). Role-Emotional and Mental Health scores were similar between CFS patients and controls.

**Table 1:** Demographics and RAND-36 results of subjects selected for the study.

		CFS Patients	Healthy Control Subjects
	**Male/Female**	0/12	0/12
	**Age (years)**	41.1±12.0	39.7±9.4
	**BMI (kg/m^2^)**	22.5±2.4	24.2±3.6
**Physical Health**	**Physical Functioning**	38.9±8.7*	95.0±1.5
	**Role-Physical**	14.8±8.5*	97.4±2.3
	**Bodily Pain**	56.9±8.0*	87.5±3.6
	**General Health**	22.5±6.4*	78.2±3.8
**Mental Health**	**Vitality**	19.2±6.4*	71.7±3.8
	**Social Functioning**	30.4±8.7*	91.0±4.1
	**Role-Emotional**	63.9±13.3	81.8±10.4
	**Mental Health**	72.0±4.6	78.7±3.8
	**Age CFS of first symptoms (years)**	31.8±3.4	N/A
	**Age of CFS diagnosis (years)**	32.6±10.7	N/A

Demographic information and RAND-36 results for CFS patients and healthy control subjects selected for DNA methylome analysis. * = p<0.05, Student’s t-test, CFS versus healthy control subjects. Data are shown as mean ± standard error of the mean, where applicable.

### Identification of differentially methylated CpG sites in CFS

After microarray normalization of the raw DNA methylation data, 327,409 probes remained for subsequent analysis. Applying the probe selection criteria, 1,192 CpG sites were identified as differentially methylated between CFS patients and healthy control subjects, corresponding to 826 genes. The list of differentially methylated CpG sites and their respective genes can be found in Table S2.

**Figure 1:**
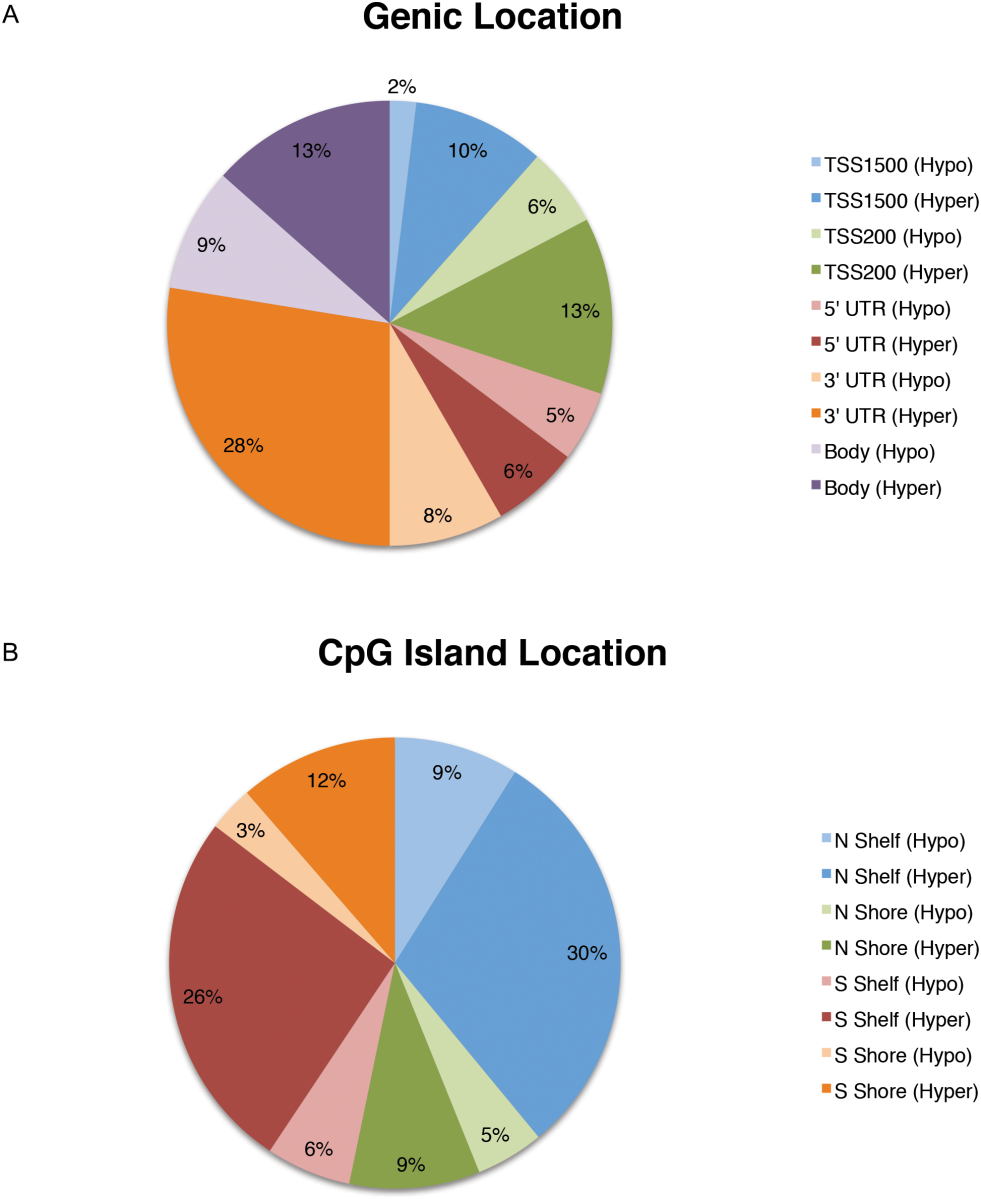
Distribution of differentially methylated regions in CFS. Distribution of hyper- and hypo-methylated CpG regions in CFS patients compared to healthy control subjects according to (**a**) genic location 1500 bp and 200 bp relative to the transcription start site (TSS), in the 5′ UTR, 3′ UTR, and within gene bodies and (**b**) location relative to CpG islands, including 2 kb upstream and downstream of CpG islands (N, S Shore respectively), and 2 kb upstream and downstream of CpG shores (N, S Shelf respectively). No significant differences were found within CpG islands.

Region-level analysis was performed by grouping probes within their respective annotations, and comparing average methylation values for all CpG sites at each location between CFS patients and healthy controls using the IMA regionswrapper function. A total of 934 differentially methylated CpG sites were found within or proximal to genes (i.e., genic locations) and 448 differentially methylated CpG sites were mapped with respect to their location relative to CpG islands (i.e. CpG island locations). As shown in Figure 1A, within genic regions, 30% of differentially methylated regions were hypomethylated and 70% were hypermethylated overall (Table S3). Differential methylation was localized predominantly to regions 1500 bp and 200 bp proximal to transcription start sites (31% in TSS1500 and TSS200) and more broadly within gene regulatory elements (78% in TSS1500, TSS200, 5′UTR and 3′UTR). Gene bodies contained 22% of differential methylation. Hypomethylated regions consisted of 8% of TSS regions, 21% of gene regulatory elements and 9% of gene bodies. Hypermethylated regions consisted of 23% of TSS regions, 57% of gene regulatory elements and 13% of gene bodies. As shown in Figure 1B, proximal to CpG islands, 23% of differentially methylated regions were hypomethylated, and 77% were hypermethylated overall. 29% of differentially methylated CpGs were located 2 kb upstream and downstream of CpG islands (N, S Shores), and 71% of differentially methylated CpGs were located 2 kb upstream and downstream of CpG shores (N, S Shelves). Within these regions, 23% of shores and shelves were hypomethylated, while 77% of CpG island shores and shelves were hypermethylated. No significant enrichment of DNA methylation differences was observed within 1^st^ exons or CpG islands.

### Validation of microarray results by pyrosequencing

**Figure 2:**
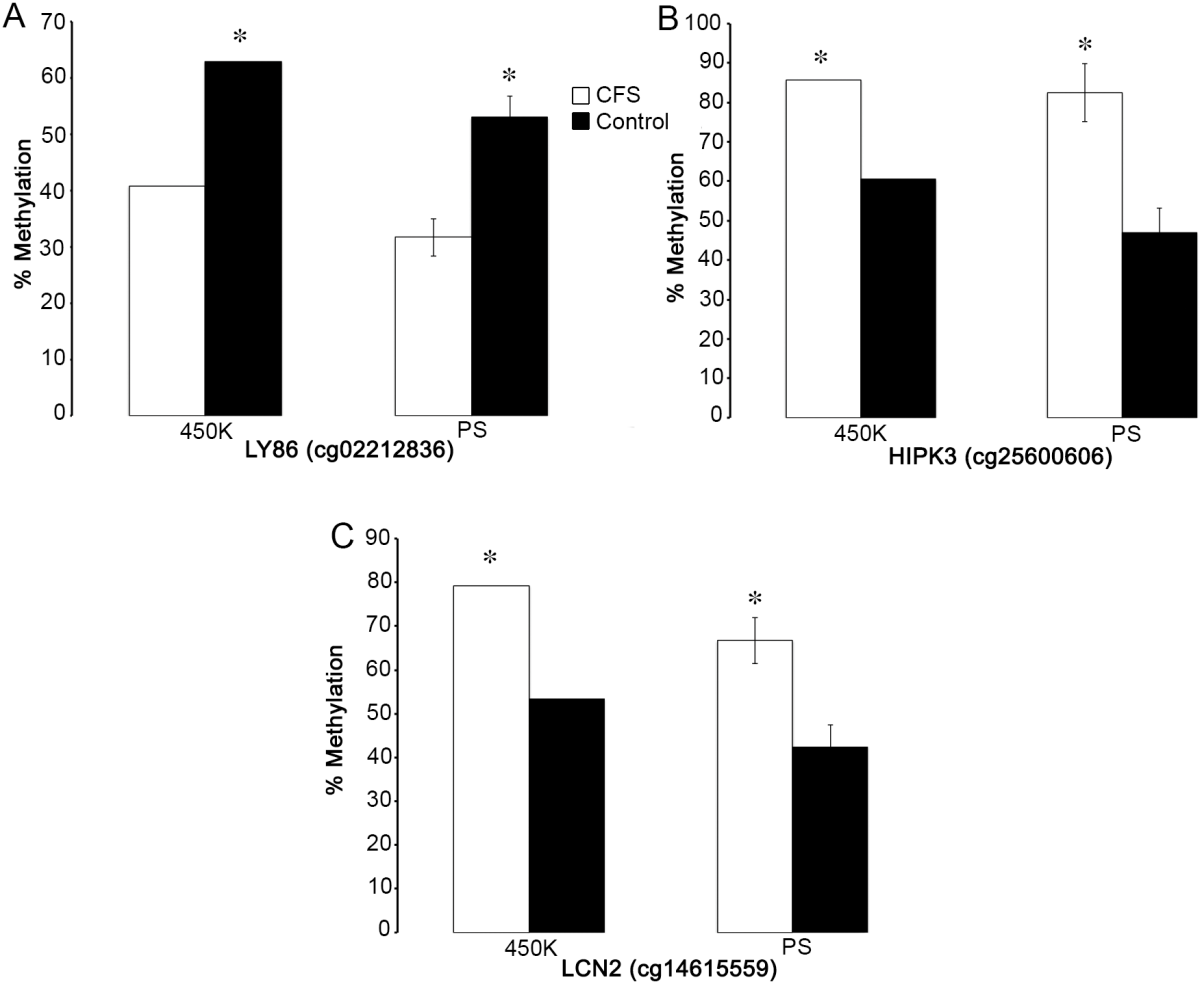
Validation of microarray data by pyrosequencing. Validation of significant methylation differences identified by microarray (450 K) by pyrosequencing (PS), showing the average methylation level of CpG sites within the following genes (probe ID, genic location): (a) LY86 (cg02212836, first exon), (b) HIPK3 (cg25600606, gene body), and (c) LCN2 (cg14615559, TSS200). * = FDR<0.05, 450 K; * = p<0.05, PS, Wilcoxon rank-sum test. Error bars represent the standard error of the mean.

Three CpG sites that were identified as significantly different on the array were selected for validation using pyrosequencing. These CpG sites were mapped to the following genes (probe ID, genic location): LY86 (cg02212836, first exon), HIPK3 (cg25600606, gene body), and LCN2 (cg14615559, TSS200). Analysis by pyrosequencing confirmed the direction of methylation differences between CFS and control subjects identified by 450 K Illumina microarray (FDR, p<0.05), with similar methylation levels detected by pyrosequencing (Wilcoxon rank-sum test, p<0.05; Figure 2).

### Gene pathway enrichment by differential DNA modification in CFS

**Figure 3:**
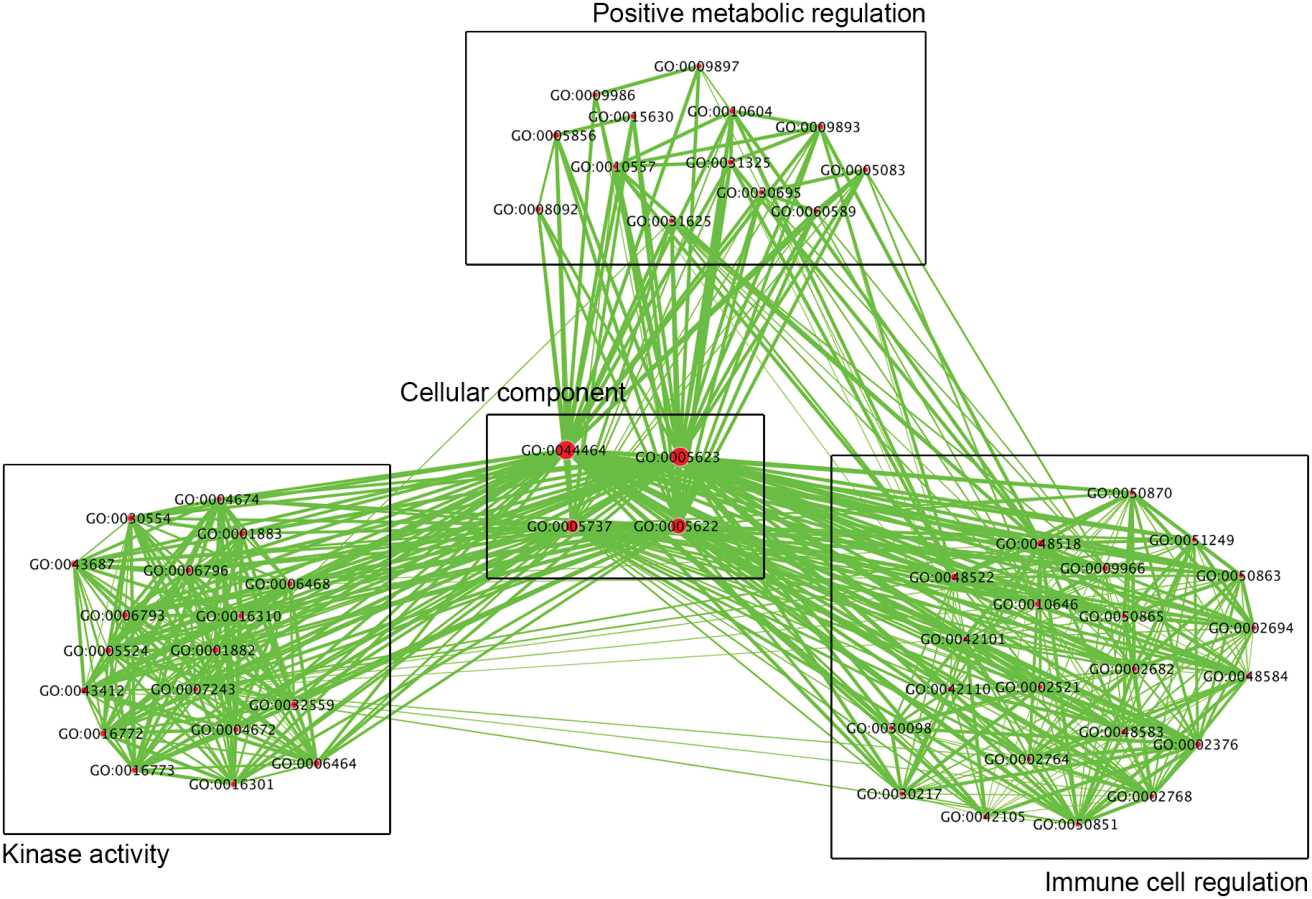
Clustering of DAVID GO results. Network map showing the clustering of DAVID GO results as produced by the Enrichment Map plugin in Cytoscape 2.8.2. Significant GO term clusters were named according to textual attributes generated by the WordCloud plugin. Node size (red circles) corresponds to the number of genes within the GO terms. Edge thickness (green lines) represents genes in common between GO terms.

To better interpret the potential functions and interactions between the genes that contained differentially methylated CpG sites, GO analysis was performed using the DAVID algorithm tool on 826 differentially methylated genes [44]. Significant GO terms representing sets of differentially methylated genes were organized into a network to identify major enriched biological themes. After grouping functionally related GO terms and applying statistical cutoffs (see Methods), 4 cluster groups were identified consisting of a total of 57 GO terms: 4 GO terms for cellular component, 13 GO terms for positive metabolic regulation, 18 GO terms for kinase activity, and 22 GO terms for immune cell regulation. Figure 3 shows the resulting network map of enriched GO terms in CFS patients compared to healthy controls, where node size corresponds to the number of genes within the GO terms and edge thickness represents genes in common between GO terms. The full list of GO terms associated with Figure 3 is found in Table S4. We also performed GO analysis with GeneMANIA [45] to compare the DAVID results with an independent algorithm. GeneMANIA analysis using the same differentially methylated gene list as above generated similar results to DAVID (Table S5).

**Figure 4:**
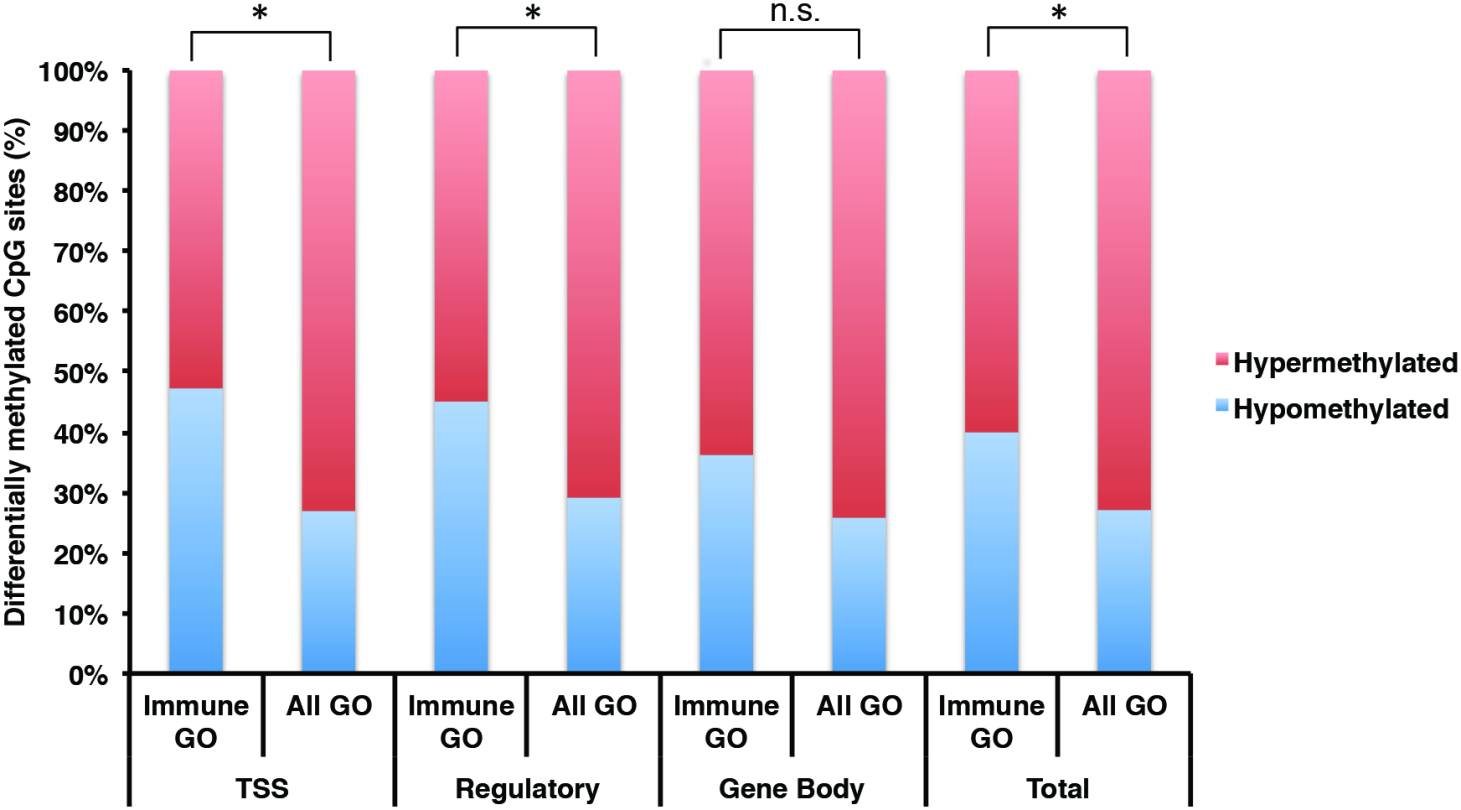
Distribution of differentially methylated sites in CFS according to GO clusters and functional relevance. Relative proportions of hyper- and hypo-methylated CpG sites between CFS patients and healthy control subjects for genes associated with the immune cell regulation cluster group (immune GO) compared to all four GO term cluster groups (all GO). Results are shown for each genic region, consisting of promoter regions within 1500 bp and 200 bp of the transcription start sites (TSS), gene regulatory elements (regulatory: TSS1500, TSS200, 5′ UTR, 3′ UTR), the coding regions of genes (gene body), as well as all regions combined (total: regulatory, gene body). * = p<0.0125, Pearson Chi-Squared Test.

Within the 4 cluster groups, 511 unique genes containing a total of 637 CpG sites were significantly hypermethylated among CFS patients compared to healthy controls, and 184 unique genes containing 237 CpGs were significantly hypomethylated. The full list of differentially methylated genes and their associated CpG sites is provided in Table S6. To examine the potential biological meaning of differentially methylated CpG sites, we determined the localization of differentially methylated CpGs in promoter regions within 1500 bp and 200 bp of transcription start sites (TSS: TSS1500, TSS200), in gene regulatory elements (regulatory: TSS1500, TSS200, 5′ UTR, 3′ UTR) and in gene coding regions (gene body). For hypermethylated genes, the proportion of differentially methylated CpGs in TSS, regulatory, and gene body regions was 73.05%, 70.83%, and 74.17%, respectively (Figure 4). For hypomethylated genes, the proportion found within each of these regions was 26.95% (TSS), 29.17% (regulatory), and 25.84% (gene body).

We next examined differential methylation of specific genes within the immune cell regulation cluster, as it showed the largest coordinated enrichment of differentially methylated gene pathways, with 22 GO terms (Figure 3; Table 2). In total, the immune cell regulation cluster contained 124 unique genes with 144 hypermethylated CpG sites and 68 unique genes with 96 hypomethylated CpG sites among CFS patients compared to healthy controls. In genes within the immune cell regulation cluster group, the proportions of hypermethylated CpGs in TSS, regulatory, and gene body were 52.73%, 54.9%, and 63.77%, respectively (Figure 4). However, the proportion of hypomethylated CpGs in TSS, regulatory, and gene body regions was 47.27%, 45.09%, and 36.23%. Compared to all 4 GO cluster groups, genes within the immune cell regulation cluster showed a significant enrichment in the relative proportion of hypomethylated CpGs in TSS and gene regulatory elements (p’s<0.0125, Pearson Chi-Square tests) but not in gene body regions. Examples of 10 immune genes hypermethylated and 10 immune genes hypomethylated at gene regulatory elements are listed in Table 3, with the full list of differentially methylated sites listed in Table S7. Overall, a greater proportion of differentially methylated CpG sites were hypomethylated among genes within all regions in the immune cell regulation cluster (40%) compared to the proportion of hypomethylated genes in all four cluster groups (27.12%), indicating a shift towards hypomethylated immune genes in CFS patients compared to healthy controls.

**Table 2:** Immune cell regulation GO cluster.

GO term	Number of Differentially Methylated Genes	p-value	Adjusted p-value (FDR)
GO:0042101∼T cell receptor complex	7	5.71E-06	0.0026
GO:0009966∼regulation of signal transduction	69	8.87E-06	0.0123
GO:0042105∼alpha-beta T cell receptor complex	4	3.43E-04	0.0266
GO:0002682∼regulation of immune system process	36	5.46E-05	0.0300
GO:0010646∼regulation of cell communication	73	1.84E-04	0.0500
GO:0030217∼T cell differentiation	12	1.57E-04	0.0532
GO:0048518∼positive regulation of biological process	126	1.49E-04	0.0578
GO:0002376∼immune system process	67	2.63E-04	0.0593
GO:0002521∼leukocyte differentiation	17	3.11E-04	0.0602
GO:0048583∼regulation of response to stimulus	39	2.93E-04	0.0610
GO:0050863∼regulation of T cell activation	16	2.54E-04	0.0625
GO:0048522∼positive regulation of cellular process	114	4.25E-04	0.0714
GO:0002694∼regulation of leukocyte activation	19	5.08E-04	0.0758
GO:0002768∼immune response-regulating cellsurface receptor signaling pathway	9	5.41E-04	0.0764
GO:0042110∼T cell activation	16	5.74E-04	0.0770
GO:0050870∼positive regulation of T cell activation	12	6.36E-04	0.0776
GO:0050851∼antigen receptor-mediatedsignaling pathway	8	6.08E-04	0.0777
GO:0030098∼lymphocyte differentiation	14	8.14E-04	0.0904
GO:0051249∼regulation of lymphocyte activation	17	1.03E-03	0.0951
GO:0048584∼positive regulation ofresponse to stimulus	23	9.73E-04	0.0958
GO:0002764∼immune response-regulatingsignal transduction	10	9.07E-04	0.0963
GO:0050865∼regulation of cell activation	19	9.57E-04	0.0977

GO terms associated with the immune cell regulation cluster for genes differentially methylated in CFS patients compared to healthy control subjects.

**Table 3:** Immune cell regulation GO cluster genes.

GeneName	Gene Accession Number	Description	Number ofDifferentiallyMethylatedCpGs	Genic Region	Methylationstatus (CFS-CTL)
ATG7	NM_001136031/NM_001144912/NM_006395	ATG7 autophagy related 7homolog (S. cerevisiae)	1	TSS1500	hypermethylated
BCL10	NM_003921	B-cell CLL/lymphoma 10	1	TSS200	hypermethylated
CD83	NM_001040280/NM_004233	CD83 molecule	1	3′ UTR	hypermethylated
FCER2	NM_002002	Fc fragment of IgE, low affinity II,receptor for (CD23)	1	TSS1500	hypermethylated
HLA-H	NR_001434	major histocompatibility complex,class I, H (pseudogene)	1	TSS200	hypermethylated
IL19	NM_153758	interleukin 19	1	TSS1500	hypermethylated
IL1RL1	NM_016232	interleukin 1 receptor-like 1	1	TSS1500	hypermethylated
LAIR1	NM_002287/NM_021706	leukocyte-associatedimmunoglobulin-like receptor 1	1	TSS1500	hypermethylated
TNFSF13B	NM_001145645/NM_006573	tumor necrosis factor (ligand)superfamily, member 13b	1	TSS1500	hypermethylated
TREM2	NM_018965	triggering receptor expressedon myeloid cells 2	1	TSS200	hypermethylated
CD3D	NM_000732/NM_001040651	CD3d molecule, delta(CD3-TCR complex)	4	TSS1500	hypomethylated
CD3E	NM_000733	CD3e molecule, epsilon(CD3-TCR complex)	1	5′ UTR	hypomethylated
CD3G	NM_000073	CD3g molecule, gamma(CD3-TCR complex)	4	5′ UTR/1st Exon	hypomethylated
CD96	NM_005816/NM_198196	CD96 molecule	1	TSS200	hypomethylated
FASLG	NM_000639	Fas ligand (TNF superfamily,member 6)	1	TSS200	hypomethylated
HLA-E	NM_005516	major histocompatibilitycomplex, class I, E	2	3′ UTR	hypomethylated
ICOS	NM_012092	inducible T-cell co-stimulator	1	5′ UTR/1st Exon	hypomethylated
LAX1	NM_001136190/NM_017773	lymphocyte transmembraneadaptor 1	2	5′ UTR/1st Exon	hypomethylated
LCK	NM_005356	lymphocyte-specificprotein tyrosine kinase	2	5′ UTR	hypomethylated
LY9	NM_001033667/NM_002348	lymphocyte antigen 9	1	TSS200	hypomethylated

Examples of genes within the immune cell regulation cluster differentially methylated at gene regulatory elements in CFS patients compared to healthy control subjects.

## Discussion

CFS is a serious and debilitating disorder characterized by a constellation of physical symptoms and is known to occur following infection [46]. This study focused on a subgroup of patients that reported sudden, infectious onset of their CFS and were required to have post-exertional malaise lasting >24 hours, cognitive impairment and at least 2 of these three RAND-36 scores; vitality ≤35, social functioning ≤62.5, role-physical functioning ≤50. As shown in Table 1, all the physical and social concepts measured were significantly different between patients and controls whereas two of the mental health subscales were not different. The fact that physical and social function are seriously impacted in CFS and detected using health outcome quality of life scales has been a highly consistent finding [47]. This also indicates that even though the sample size for this study was small, it was carefully selected and likely representative of sudden onset CFS.

This study had several major findings. We identified differentially methylated CpG sites in PBMCs of female CFS patients compared to healthy controls, who were selected to control potential age-, obesity-, and medication-related influences on epigenetic profiles. We found significant differences in DNA methylation between CFS patients and healthy controls at 1,192 CpG sits in 826 genes overall, with differential DNA methylation present across promoters, gene regulatory elements and within coding regions of genes. GO terms related to cellular processes, positive metabolic regulation, kinase activity and the immune response were enriched among CFS patients; similar GO terms were found using quantitative trait analysis of gene expression in a distinct CFS patient population [48]. Organizing GO results into a network indicated an overrepresentation of terms related to immune cell regulation, consistent with previous studies examining functional changes in immune profiles associated with CFS [7], [11], [12]. Differentially methylated genes related to immune cell regulation showed an increased proportion of hypomethylated CpG sites among CFS patients, particularly in promoters and in gene regulatory elements, relative to the distribution of differentially methylated sites in the network as a whole.

Differential methylation of gene regulatory elements is classically associated with alterations in gene expression [49]. In genes within the immune cell regulation cluster, we found a number of differentially methylated CpGs in gene regulatory elements associated with the immune response. These data are consistent with previous observations of a Th1- to Th2-mediated immune response shift in CFS [7], [50]. A number of studies have linked altered immune system function with CFS [11], [12] and have found that gene expression differences in some immune genes in PBMCs can be used to distinguish between CFS patients and healthy controls [51], [52]. Transcriptional profiling studies have indicated perturbations in T-cell [53]–[55] and B-cell activation [56] and dysregulation in gene expression broadly related to immune responses [57], changes that parallel other studies showing altered production of interleukin and interferon cytokines in CFS patients [50]. Consistent with these observations, we also observed changes in DNA methylation within a number of genes known to regulate the adaptive immune response. For example, BCL10, FCER2, and IL1RL1/ST2 were hypermethylated among the genes in the immune GO cluster (Table 3). BCL10 is a known activator of the NFkB pathway [58], [59], which aligns with the immune response differences seen in CFS. FCER2 has been implicated in B-cell activation and has shown expression differences in B cells infected by the Epstein-Barr virus [60]. Lastly, IL1RL1/ST2 is a known Th2 cell marker [61] which activates MAP kinases [62]. At this time, we do not know whether these epigenetic modifications are indicative of homeostatic compensations or are the result of an adaptive immune response to environmental exposures. These results are consistent with DNA methylation as a potential mechanism of long-term effects on the regulation of gene expression and noted long-term alterations in gene expression observed in previous studies of CFS [5], [11], [12], [63].

Hypomethylated CpG sites were significantly enriched in promoters and gene regulatory elements of genes related to immune signaling. It is possible that these immune genes may show increased transcript abundance or increased transcriptional potential, at least among the CFS group selected in this study, as promoter hypomethylation is generally associated with an increase in gene expression [49]. CFS symptoms are known to worsen causing significant debility after exertion [64], concomitant with an increase in inflammatory gene expression [65]. Such data suggest that latent alterations in immune system function may be ‘unmasked’ during challenge conditions. Future studies aimed at examining the relationship between epigenetic and gene expression profiles of CFS patients under both basal and challenge conditions will be informative in elucidating the relationship between epigenetic mechanisms and functional changes in gene regulation in CFS.

Epigenomic studies aimed at characterizing the properties of immune cells have documented distinct profiles between cellular subtypes [20], [66]. Epigenetic disruptions in immune cells can lead to chronic impairment in the function of important immune cell subtypes [67]–[69], and are implicated in various disorders with an immunological component, such as chronic depression, lupus, and rheumatoid arthritis [20], [70], [71]. Although the data collected in this study do not rule out the possibility of differential contributions of epigenetic variation of specific subtypes of immune cells within our mixed PBMC cell population, it is worth noting that the relevant cell populations affected in CFS remain unknown. Nevertheless, our data provide evidence that epigenetic variation in CFS may be distinct, at least in part, from related disorders with an immunological component. For example, epigenomic analysis of whole blood in fibromyalgia (FM) patients indicated differential methylation in genes associated with structural and nervous system development and neuron differentiation [72]. Although FM has a similar symptom profile compared to and is comorbid with CFS [73], our results contribute to the growing number of studies indicating biological differences between the two diseases [74].

Although the immune system showed most changes in DNA methylation, we also found an enrichment in gene sets linked to cellular components, kinase activity, and positive metabolic activity, supporting previous data indicating differences in the expression of genes associated with cellular metabolism and oxidative stress in PBMCs from CFS patients [48], [51], [52]. In CFS, disruptions of pathways related to structural components of the cell and metabolic proteins via cellular stress are linked to altered functional outcomes [75]. For example, increased rates of apoptosis in leukocytes have been observed in CFS, which could be explained through elevations in protein kinase activity [76]. However, the specific role of kinases in CFS is not well understood and more research is required to elucidate their contribution to CFS pathology.

The results of this study do not indicate whether these observed epigenetic differences are a cause or a consequence of CFS. However, we provide evidence suggesting a potential role for epigenetic alterations in the pathophysiology of CFS. We controlled for genetic polymorphisms known to overlap with array probes used to directly quantify DNA methylation. It remains possible that contributions of genetic differences that are distal from probe sites may contribute to differences in epigenetic signaling [77]. An analysis of genome-wide genetic differences in a CFS cohort identified 65 single nucleotide polymorphisms (SNPs) associated with CFS located in 9 chromosomes, indicating genetic differences across the genome may contribute to CFS pathology [78]. However, the fact that genetic variations in only two of these SNPs were linked to changes in gene expression suggests that other mechanisms of gene regulation are likely involved in CFS. Epigenetic mediation of the connection between genotype and phenotype has recently been proposed [79], and these relationships may be worth investigating in assessments of disease and disease risk. Examining epigenetic modifications in CFS is of particular interest as epigenetic changes can exert long-term effects on gene expression and are potentially amenable to therapeutic intervention [22]. For example, therapeutic interventions targeting epigenetic mechanisms in cancer have shown some success in altering inflammatory pathways [80]. In particular, 5-azacytidine, a DNA hypomethylating drug, has been shown to alter DNA methylation of immune pathways in non-small cell lung cancer cell lines, including genes in the viral defense, stress response, and human leukocyte antigen (HLA) Class I processing pathways [80]. HLA-E and HLA-H, which are a part of the HLA Class I processing pathway, were among those that showed the greatest differences in CFS in the immune gene GO cluster (Table 3) and could serve as potential markers for future targeted therapeutic studies in CFS. Longitudinal studies examining differences in the epigenomes of CFS patients in the context of variation over time and with CFS symptomatology would help to identify these interactions and determine the stability of the epigenomic differences that were observed in this study.

## Conclusions

To our knowledge, this is the first study that has explored genome-wide epigenetic changes associated with CFS. Network analysis of enriched GO terms associated with differentially methylated genes identified GO clusters related to cell structure and function, with the largest cluster related to the immune response. Gene regulatory elements within the immune cluster were significantly hypomethylated relative to the network overall. These data are consistent with previous evidence of immunological dysregulation in CFS and implicate the involvement of DNA modifications in CFS pathology.

## Supporting Information

Table S1 **Immunomodulatory medications and medications with known effects on epigenetic mechanisms used to exclude subjects from the study.**
(XLSX)

Table S2 **CpG sites identified as differentially methylated in CFS patients compared to healthy controls.**
(XLSX)

Table S3 **Region-level analysis of differentially methylated genes grouped by location in CFS patients compared to healthy controls.**
(XLSX)

Table S4 **Significant GO terms and their cluster groups in CFS compared to healthy controls as determined by DAVID and Enrichment Map.**
(XLSX)

Table S5 **Significant GO terms in CFS compared to healthy controls as determined by GeneMANIA.**
(XLSX)

Table S6 **CpG sites in cluster group genes identified as differentially methylated between CFS patients and healthy controls.**
(XLSX)

Table S7 **CpG sites in genes within the immune cell regulation cluster identified as differentially methylated between CFS patients and healthy controls.**
(XLSX)
